# Intensity-Modulated Proton Therapy (IMPT) Treatment of Angiosarcoma of the Face and Scalp

**DOI:** 10.14338/IJPT-D-20-00048.1

**Published:** 2021-06-25

**Authors:** Ashley Hunzeker, Daniel W. Mundy, Jiasen Ma, Trey C. Mullikin, Robert L. Foote

**Affiliations:** 1 Department of Radiation Oncology, Mayo Clinic, Rochester, MN, USA

**Keywords:** Total scalp, intensity-modulated proton therapy (IMPT), angiosarcoma, Monte Carlo (MC)

## Abstract

**Purpose:**

To successfully plan and treat a patient with diffuse angiosarcoma involving the face and scalp with intensity-modulated proton therapy (IMPT) before surgical resection.

**Materials and Methods:**

A patient presented to the radiation oncology department for preoperative treatment of an angiosarcoma diffusely involving the face and scalp. A 4-field IMPT technique was used to create a homogeneous dose distribution to the entire target volume while sparing underlying critical structures from toxicity and low-dose spread. A custom Monte Carlo optimizer was necessary to achieve treatment goals. Biological dose was evaluated with a linear energy transfer–based biological enhancement model. Robustness criteria were evaluated per department standard. The patient was successfully planned and treated according to clinical goals.

**Results:**

The patient successfully completed the course of IMPT and was able to undergo surgical resection. Pathology indicated no presence of angiosarcoma.

**Conclusion:**

IMPT using a custom Monte Carlo optimizer is a suitable radiation therapy treatment option for patients with diffuse angiosarcoma of the scalp and face.

## Introduction

Total scalp irradiation presents significant challenges. Although treatment techniques for total scalp irradiation have been studied and implemented, there are treatment limitations, including field matching, dose inhomogeneity, target coverage, and unacceptable organs at risk (OARs) dose. Able et al [1] described the technique of matching 6 main electron fields with successful ocular structure sparing; however, difficulty in junction matching and general dose inhomogeneities as great as ±70% were observed [1]. Akazawa [2] and Tung et al [3] reported an improved scalp treatment technique using 2 lateral electron fields and parallel opposed lateral photon fields. Both studies demonstrated improved dose uniformity, but field junction feathering was still problematic [2, 3]. Several researchers have also explored the use of intensity-modulated radiation therapy and volumetric modulated arc therapy, noting improved planning target volume coverage and dose homogeneity; however, an increase in dose to nearby OARs, including the lenses, brain, and orbits, was undesirable [4–6]. In addition, each of the aforementioned techniques required some type of custom total scalp bolus that was time consuming to create and presented setup uncertainties for treatment delivery.

To our knowledge, no current treatment technique has entirely solved the complexities of total scalp irradiation. Moreover, there have been no reports of curative-intent treatment of total scalp and face. In this report, we describe the planning and treatment of a patient who presented with an angiosarcoma diffusely involving the scalp and face and requiring total scalp and face preoperative radiotherapy using intensity-modulated proton therapy (IMPT) and a custom Monte Carlo (MC) optimizer [7, 8].

## Patient and Methods

The authors sought approval from the institutional review board at the referenced medical facility to publish this case report, which was considered exempt from research.

**Figure 1.**
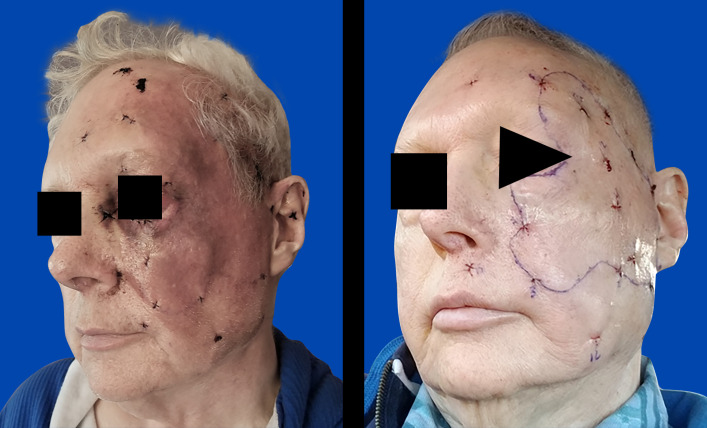
The pre-chemotherapy erythematous rash after mapping biopsies (left). The resolved rash after chemotherapy (right).

The patient was a 67-year-old man who presented with erythema of the face and scalp. Mapping biopsies revealed diffuse involvement of the scalp and face with angiosarcoma. He was treated with 3 cycles of gemcitabine and docetaxel, which completely resolved the left facial erythematous rash; however, repeated mapping biopsies were still positive for disease (Figure 1). This chemotherapy regimen was then followed by concurrent weekly paclitaxel and IMPT. The patient then underwent left hemi-facial and scalp resection with delayed autologous flap reconstruction.

### Computed Tomography Simulation

The patient underwent computed tomography simulation in the head-first supine position. The patient's hands were positioned on his abdomen holding a ring. A custom headrest was created using multiple Klarity cushions (Klarity Medical, Heath, Ohio), and a moldable Truguard bite block (Bionix Radiation Therapy, Toledo, Ohio) was fashioned to maintain head position. A 5-point aquaplast mask (Orfit Industries, Wijnegem, Belgium) was then molded over the head, neck, and shoulder regions and anchored to the mouth guard to maintain alignment and increase reproducibility. The patient was scanned at 1 mm increments from the top of the head through the carina; the images were then uploaded into the Eclipse treatment planning system (TPS; Varian Medical Systems, Palo Alto, California).

### Target Delineation and Contouring

**Figure 2.**
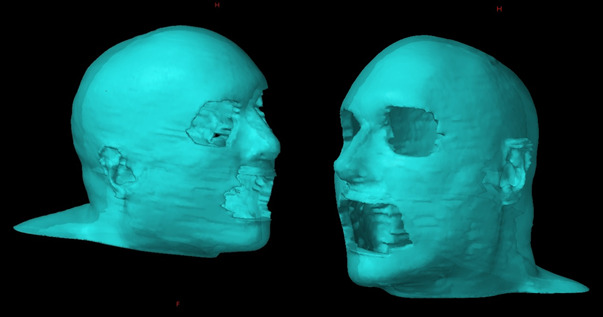
3-dimensional rendering of the clinical target volume.

The clinical target volume (CTV) was defined by the physician and included the entire cutaneous tissues of the scalp and face, excluding the eyelids and lips, inferiorly to the level of the thyroid notch and C5 (Figure 2). Several OARs were delineated, including the brain, brainstem, lenses, eyes, retinas, lacrimal glands, bilateral auditory structures, pharyngeal constrictors, spinal cord, cricopharyngeal inlet, optic chiasm, optic nerves, larynx, lips, mandible, nasal cavity, oral cavity, bilateral parotid and submandibular glands, pituitary gland, and thyroid gland.

### Treatment Planning

The radiation oncologist prescribed 60 Gy (relative biological effectiveness [RBE] 1.1) to be delivered to the CTV in 30 fractions over 6 weeks using IMPT (Table). Priority was placed on sparing brain, eyes, retinas, and lacrimal glands (Table).

**Table.** Desired constraints for the most critical organs at risk.^a^

**Structure**	**Parameter**	**Desired objective**	**Achieved objective**
Clinical target volume 6000	D_95%_	100% (60 GyRBE)	96.1% (57.36 GyRBE)
	V_100%_	95%	81.4%
Brain, GyRBE	D_0.01cm_^3^	56	68.3
	Mean	10	4.0
Eye (right; left), GyRBE	Mean	20	8.3; 9.7
Retina (right; left), GyRBE	Mean	20	8.2; 11.4
Lacrimal glands (right; left), GyRBE	Mean	10	17.7; 24.6
	D_0.03cm_^3^	25	26.2; 38.9
Parotid glands (right; left), GyRBE	Mean	20	28.1; 25.8
Submandibular glands (right; left), GyRBE	Mean	35	13.4; 28.1
Lips, GyRBE	Mean	20	29.4
	D_0.03cm_^3^	50	56.2

**Abbreviations:** D_95%_, 95% of the volume received specified dose; V_100%_, volume receiving 100% of prescribed dose; D_0.01cm_^3^, 0.01cm^3^ received specified dose or more; D_0.03cm_^3^, 0.03cm^3^ received specified dose or more.

a This table contains a select number of the structures and objectives used for this plan. Structures not listed include the pituitary, brainstem, spinal cord, auditory structures, optic chiasm, bilateral optic nerves, oral cavity, nasal cavity, mandible, thyroid, pharyngeal constrictors, cricopharyngeal inlet, and larynx. Dose to these structures was either not significant or pertinent to this case report.

Planning OAR volumes were added to the brainstem and spinal cord with margins of 2 mm and 5 mm, respectively. The CTV was expanded by 2 mm into an optimization target volume to improve coverage and plan robustness. The optimization target volume was cropped away from the lips and lacrimal glands by 1 cm, and OAR optimization structures were created for the bilateral lacrimal, parotid and submandibular glands.

A 4.5-cm range shifter with a spot size of approximately 8 mm in air at isocenter was used to treat the superficial target using a multifield optimization technique. A 4-field approach was designed, including a right posterior oblique, a right anterior oblique, a left anterior oblique, and an anterior superior oblique. Field-specific scanning target volumes (STVs) were established for each field to dictate the boundaries for spot placement accounting for setup and range uncertainties. Each field was only allowed to treat the CTV in closest proximity without crossing midline to maximize OAR sparing.

First attempts at plan optimization in the Eclipse TPS were unsuccessful. Areas of the target were grossly undercovered, while others exceeded prescription dose by approximately 10% to 20%. Critical OAR doses were also unacceptable. After a discussion with the consulting medical physicist, the team elected to optimize the plan using a custom MC optimizer [7, 8] developed at Mayo Clinic. The custom optimizer was designed to consider linear energy transfer and associated RBE during optimization and generated MC-calculated physical and linear energy transfer –weighted dose maps for evaluation [7, 8]. The MC dose calculation used in the optimizer is more accurate at very shallow depths than the analytic dose calculation algorithms used in most treatment planning software, making it particularly suited to this case [7, 8].

No changes to the treatment machine setup, field arrangement, or STV configuration were made. The plan was robustly optimized to maintain coverage with isocenter shifts up to 3 mm in all cardinal directions and range uncertainty up to 3%.

**Figure 3.**
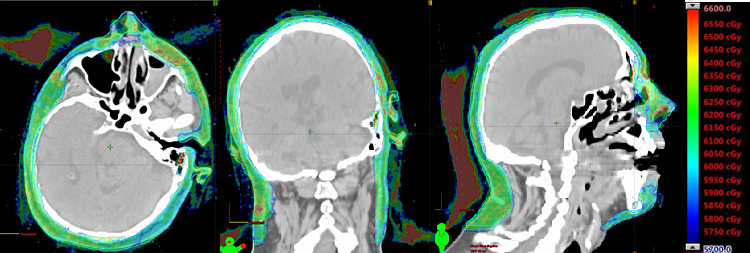
Axial, coronal, and sagittal views of dose distribution.

**Figure 4.**
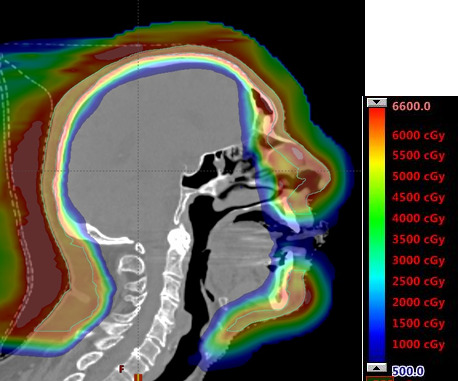
Sagittal view of dose profile. The intensity-modulated proton therapy plan was successfully used to treat the target volume while limiting 5 GyRBE to the underlying brain and organs at risk.

The final plan achieved a fairly homogeneous dose distribution and OAR constraints (Figure 3, Table). The plan nearly achieved coverage goals. The areas where dose was compromised (skin of the bridge of the nose between eyes, skin near lacrimal glands, and skin near lips) were considered low risk for subclinical disease, necessary to achieve OAR constraints, and acceptable per the radiation oncologist. The mean brain dose was observed at just over 4 GyRBE with a maximum dose of 68 GyRBE to 0.01 cm^3^ (Figure 4). Though the maximum brain dose exceeded the desired constraint, only 0.05 cm^3^ of the brain was receiving 56 GyRBE or more and was accepted by the radiation oncologist. The eyes, retinas, and submandibular and parotid glands were safely spared. The constraints for the lacrimal glands and lips were not achieved despite decreasing the dose to the CTV in the areas of closest proximity.

The RBE dose was also reviewed by the radiation oncologist. Areas of enhancement were noted in the skull and were consistent with field arrangement and STV configuration. Enhancement in critical structures was reviewed and determined to be in a safe location or was small enough in volume to avoid severe side effects.

Plan robustness was evaluated with 3-mm isocenter shifts and 3% range variation. A minimum coverage of 91.6% of the dose treating 91.6% of the target was observed with an inferior shift. Since there were excellent bony landmarks (skull, skull base, orbits, maxilla, and mandible) to verify treatment position, the setup uncertainties were not of significant concern, and the plan robustness was accepted [9].

### Plan Verification

Department protocols dictate weekly computed tomography verification scans based on review of our initial experience with head and neck proton patients [10]. One replan was completed at week 2 for this patient based on decreased target coverage associated with weight loss and positional shifts.

## Results

**Figure 5.**
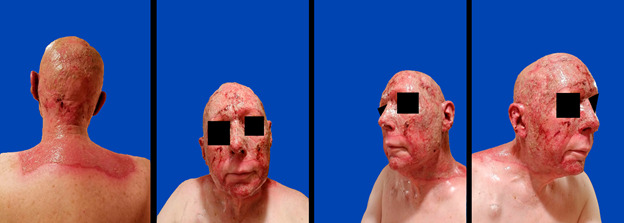
The patient experienced grade 3 dermatitis after completing radiation therapy.

**Figure 6.**
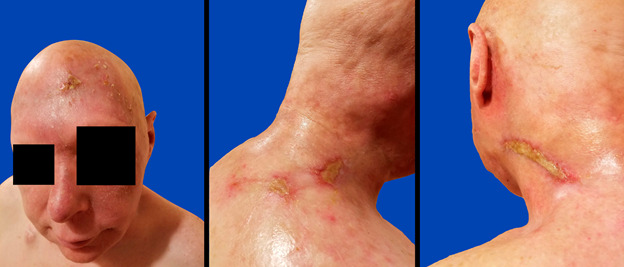
Radiation dermatitis mostly resolved 103 days after radiation therapy treatment.

Acute treatment-related toxicity was prospectively scored using Common Terminology Criteria for Adverse Events version 5.0 (National Cancer Institute Bethesda, MD). Prior to beginning treatment, the patient's Eastern Cooperative Oncology Group (ECOG) performance status was 0, and he had grade 1 anxiety related to the diagnosis and anticipated treatment with chemotherapy, IMPT, and surgery. At the end of concurrent weekly paclitaxel and IMPT, the patient's ECOG performance status had declined to 2, with a Karnofsky performance status of 70. He developed grade 3 radiation dermatitis and grade 3 dehydration, requiring a 9-day hospitalization for intravenous fluids and aggressive skin care, during which he completed his treatment (Figure 5). He developed grade 2 alopecia, oral pain, pain of skin and oral mucositis (anterior floor of mouth and tip of tongue) requiring narcotic pain medication, and grade 1 fatigue. There were no treatment interruptions. He underwent surgery 103 days after completing IMPT. No residual angiosarcoma was noted in the left hemi-facial and scalp specimen. The surgery was delayed due to delayed healing of the dermatitis (Figure 6). At 109 days after completing IMPT, the patient's ECOG performance status was 1, and the Karnofsky performance status was 80. He had grade 3 facial nerve dysfunction due to surgery, grade 2 alopecia and dry eyes, grade 1 dermatitis, skin pain (due to surgery), and watering eyes. There was no dry mouth or altered taste. The patient was alive and free of disease 26 months from the date of diagnosis and 14.5 months after completing all treatment.

## Discussion

Traditional radiation therapy techniques have benefits and limitations when treating the entire scalp. However, to our knowledge, no technique has included the entire face in addition to the scalp, nor has any technique been successfully used to eradicate these limitations. Use of IMPT with the Eclipse TPS was not successful in this case. The optimizer was unable to deliver a homogeneous dose distribution to the target while sparing underlying tissues. However, the custom MC IMPT optimizer effectively treated the large target volume to the acceptable dose and created a homogeneous dose profile that eliminated cold and hot areas. The entire treatment area was encompassed in a single 4-field arrangement around 1 isocenter. In addition, the custom MC IMPT optimizer successfully spared the underlying brain (Figure 4). Only 20% of the brain received 5 GyRBE or more, successfully sparing the organ from unwanted acute and potentially late side effects. Other OARs, including the ocular structures and salivary glands, were within tolerance as well. The custom MC IMPT plan was robust to daily uncertainties, and only after the patient had lost weight was a replan necessary. Notably, the patient did not have xerostomia; however, the care team noted prolonged time for the radiation dermatitis to heal. The increased severity of acute skin toxicity may have been related to the treatment volume, concurrent chemotherapy, and biological dose effects not appreciated in our optimization.

## Conclusion

This case study presented a unique set of challenges for the radiation oncologist, medical physicist, and medical dosimetrist. While the previous literature suggested possible treatment techniques, the care team sought to develop a technique that could provide homogeneous and total dose coverage while minimizing dose to the underlying brain and other OARs. Though this was successful, a custom MC optimizer was required to produce a homogeneous plan with as low as reasonably achievable OAR doses. Some limitations with this IMPT technique existed. Due to the need for an in-house optimizer, it would currently be difficult to execute this treatment at other proton therapy facilities. Moreover, because of our close proximity to an inpatient hospital, the patient was able to complete treatment without interruption despite a 9-day hospitalization for supportive care. In conclusion, we find that IMPT is a technically feasible method for treating malignancies diffusely involving the face and scalp.
